# (*RS*)-1-[5-(2-Chloro­prop­yl)indolin-1-yl]ethanone

**DOI:** 10.1107/S1600536810050476

**Published:** 2010-12-08

**Authors:** Xue-Mei Yang

**Affiliations:** a Department of Chemistry, Guangdong Medical College, Dongguan 523808, People’s Republic of China

## Abstract

In the title compound, C_13_H_16_ClNO, the acetyl­indoline moiety is roughly planar (r.m.s. deviation = 0.0048 Å). The chloro­propyl group is out of the plane and is statistically disordered over two positions. Indeed, the Cl and CH_3_ groups located on the stereogenic carbon exchange with each other. The whole crystal is a racemate. Non-classical C—H⋯O hydrogen bonds between symmetry-related benzene rings stabilize the crystal structure.

## Related literature

The title compound was synthesized as an inter­mediate in the search for a new synthetic route to silodosin, an adrenoceptor antagonist, see: Asselin *et al.* (2000 ▶); Bremner *et al.* (2000 ▶); Elworthy *et al.* (1997 ▶); Sorbera *et al.* (2001 ▶). For related structures, see: Moreno *et al.* (1998 ▶); Wang *et al.*(2007 ▶).
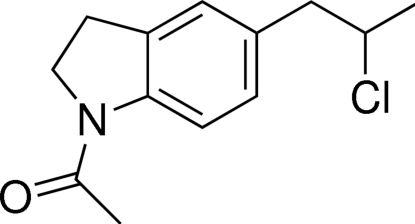


## Experimental

### 

#### Crystal data

C_13_H_16_ClNO
*M* *_r_* = 237.72
Triclinic, 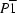

*a* = 6.9041 (5) Å
*b* = 8.4887 (6) Å
*c* = 10.6463 (7) Å
α = 76.423 (1)°
β = 86.955 (1)°
γ = 89.969 (1)°
*V* = 605.61 (7) Å^3^
*Z* = 2
Mo *K*α radiation
μ = 0.29 mm^−1^
*T* = 173 K
0.46 × 0.41 × 0.22 mm

#### Data collection

Bruker AXS SMART 1000 CCD diffractometer
Absorption correction: multi-scan (*SADABS*; Sheldrick, 2008*a* ▶) *T*_min_ = 0.877, *T*_max_ = 0.938
4719 measured reflections
2343 independent reflections
1915 reflections with *I* > 2σ(*I*)
*R*_int_ = 0.016

#### Refinement

*R*[*F* ^2^ > 2σ(*F* ^2^)] = 0.041
*wR*(*F* ^2^) = 0.113
*S* = 1.07
2343 reflections
163 parameters
5 restraints
H-atom parameters constrained
Δρ_max_ = 0.35 e Å^−3^
Δρ_min_ = −0.19 e Å^−3^

### 

Data collection: *SMART* (Bruker, 2001 ▶); cell refinement: *SAINT-Plus* (Bruker, 2003 ▶); data reduction: *SAINT-Plus*; program(s) used to solve structure: *SHELXTL* (Sheldrick, 2008*b* ▶); program(s) used to refine structure: *SHELXL97* (Sheldrick, 2008*b* ▶); molecular graphics: *ORTEPIII* (Burnett & Johnson, 1996 ▶), *ORTEP-3 for Windows* (Farrugia, 1997 ▶) and *PLATON* (Spek, 2009 ▶); software used to prepare material for publication: *SHELXTL*.

## Supplementary Material

Crystal structure: contains datablocks global, I. DOI: 10.1107/S1600536810050476/dn2631sup1.cif

Structure factors: contains datablocks I. DOI: 10.1107/S1600536810050476/dn2631Isup2.hkl

Additional supplementary materials:  crystallographic information; 3D view; checkCIF report

## Figures and Tables

**Table 1:** Hydrogen-bond geometry (Å, °)

*D*—H⋯*A*	*D*—H	H⋯*A*	*D*⋯*A*	*D*—H⋯*A*
C4—H4⋯O1^i^	0.95	2.45	3.388 (2)	168
C12—H12*A*⋯O1^i^	0.96	2.44	3.388 (2)	169

Symmetry code: (i) 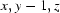.

**Fig. 1.**
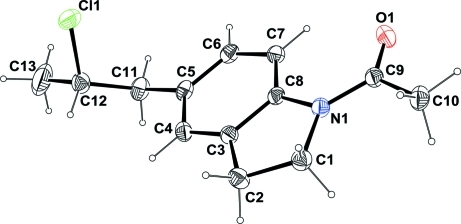
Molecular view of (I) with the atom-labelling scheme. Displacement ellipsoids are drawn at the 30% probability level. H atoms are represented as small spheres of arbitrary radii. Only one component of the disorder is shown in the figure for the sake of clarity.

**Fig. 2.**
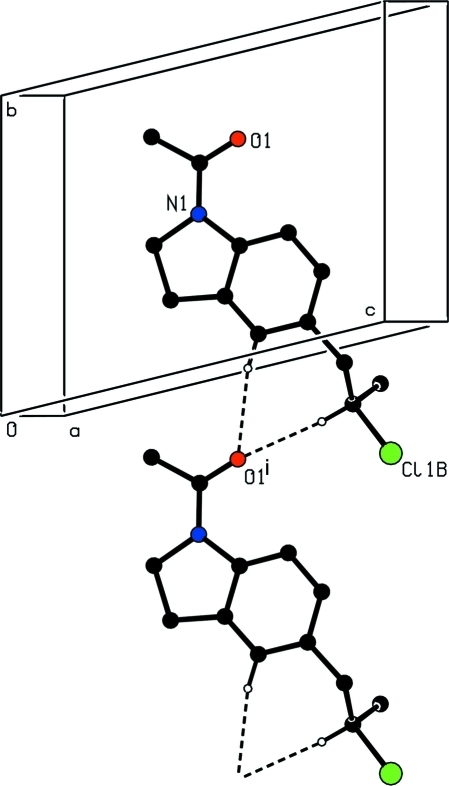
Partial packing view of compound ( I ), showing the formation of chains along [010] built from hydrogen bonds represented as dashed lines. H atoms not involved in hydrogen bonding have been omitted for clarity. [Symmetry code: (i) x, y-1, z]

## supplementary crystallographic information

### Comment

In searching for new synthetic route of silodosin, a adrenoceptor antagonist (Sorbera *et al.* 2001; Elworthy *et al.* 1997; Asselin *et al.* 2000; Bremner *et al.* 2000), we synthesized the title compound as racemic intermediate.

In the title compound, C_13_H_16_ClNO, the acetylindoline moiety is mainly planar with the largest deviation from the plane being 0.0076 (14)Å at C2. The chloropropane being out of the plane with the C12 atom located 1.0254 (0.0028)Å above the plane (Fig. 1). The chloropropane moiety is statistically disordered over two positions. Indeed, the Cl and CH_3_ located on the stereogenic carbon exchange each other. The geometry within the 1-acetylindoline fragment compares well with related structures as 1-acetylindoline (Moreno *et al.*, 1998) or 1-(trifluoro)acetylindoline (Wang *et al.*, 2007).

Non-classical C—H···O hydrogen bonds (Table 1) link the molecules forming layers parallel to the (1 0 0) plane (Figure 2).

### Experimental

1 g of (*R*/*S*)-1-(1-acetylindolin-5-yl)-2-chloropropan-1-one was dissolved in 50 ml of trifluroacetic acid, and then 1.067 g of triethylsilane was added dropwise within 20 min in -5¯C. The system was stirred overnight in ambient temperature, then extra trifluroacetic acid was distilled out in reduced pressure. To the resulting oil was added 20 ml of water and 5 ml of n-hexane, and stirred for 10 min. The white precipitate was collected through filtration, washed by n-hexane and dried to get 1.24 g of the targeting product. Crystals suitable for X-ray diffraction were obtained by slow evaporation of an ethyl acetate solution. Spectroscopic analysis: ^1^H NMR (CDCl_3_,δ, p.p.m.): 1.519–1.542(d,3*H*), 2.236(s,3*H*), 2.882–3.093(t,2*H*), 3.171–3.227(t,2*H*), 4.037–4.124(t,2*H*), 4.147–4.213(t, 1H), 7.009–7.0977(s,2*H*), 8.111–8.140(d,1*H*).

### Refinement

All H atoms attached to C atoms and N atom were fixed geometrically and treated as riding with C—H = 0.95Å (aromatic), 0.98 Å (methyl), 0.99 Å (methylene) and 0.96Å (methine) with *U*_iso_(H) = 1.2*U*_eq_(Caromatic, Cmethine, Cmethylene) or *U*_iso_(H) = 1.5*U*_eq_(Cmethyl).

The Cl and CH3 substituents on the stereogenic carbon are exchanging each other and such disorder induces two configurations. Two sets of positions were defined for the atoms of this group and the site occupation factor of each conformation were refined while restraining their sum to unity and using restraints on C—C and C—Cl distances with the help of SAME and PART instructions within *SHELXL97* (Sheldrick, 2008). In the last stage of refinement, the disordered Cl and C atoms were anisotropically refined but the anistropic thermal parameters of the C atoms were restrained to have similar atomic displacement parameters within a tolerance s.u. of 0.01 Å^2^.

### Crystal data

**Table:**

C_13_H_16_ClNO	*Z* = 2
*M**_r_* = 237.72	*F*(000) = 252
Triclinic, *P*1	*D*_x_ = 1.304 Mg m^−^^3^
Hall symbol: -P 1	Mo *K*α radiation, λ = 0.71073 Å
*a* = 6.9041 (5) Å	Cell parameters from 2890 reflections
*b* = 8.4887 (6) Å	θ = 2.5–27.0°
*c* = 10.6463 (7) Å	µ = 0.29 mm^−^^1^
α = 76.423 (1)°	*T* = 173 K
β = 86.955 (1)°	Block, colourless
γ = 89.969 (1)°	0.46 × 0.41 × 0.22 mm
*V* = 605.61 (7) Å^3^	

### Data collection

**Table:**

Bruker AXS SMART 1000 CCD diffractometer	2343 independent reflections
Radiation source: fine-focus sealed tube	1915 reflections with *I* > 2σ(*I*)
graphite	*R*_int_ = 0.016
ω scans	θ_max_ = 26.0°, θ_min_ = 2.0°
Absorption correction: multi-scan (*SADABS*; Sheldrick, 2008)	*h* = −8→8
*T*_min_ = 0.877, *T*_max_ = 0.938	*k* = −10→10
4719 measured reflections	*l* = −13→13

### Refinement

**Table:**

Refinement on *F*^2^	Primary atom site location: structure-invariant direct methods
Least-squares matrix: full	Secondary atom site location: difference Fourier map
*R*[*F*^2^ > 2σ(*F*^2^)] = 0.041	Hydrogen site location: inferred from neighbouring sites
*wR*(*F*^2^) = 0.113	H-atom parameters constrained
*S* = 1.07	*w* = 1/[σ^2^(*F*_o_^2^) + (0.0478*P*)^2^ + 0.2707*P*] where *P* = (*F*_o_^2^ + 2*F*_c_^2^)/3
2343 reflections	(Δ/σ)_max_ = 0.002
163 parameters	Δρ_max_ = 0.35 e Å^−^^3^
5 restraints	Δρ_min_ = −0.19 e Å^−^^3^

### Special details

**Table:**

Geometry. All esds (except the esd in the dihedral angle between two l.s. planes) are estimated using the full covariance matrix. The cell esds are taken into account individually in the estimation of esds in distances, angles and torsion angles; correlations between esds in cell parameters are only used when they are defined by crystal symmetry. An approximate (isotropic) treatment of cell esds is used for estimating esds involving l.s. planes.
Refinement. Refinement of *F*^2^ against ALL reflections. The weighted *R*-factor *wR* and goodness of fit *S* are based on *F*^2^, conventional *R*-factors *R* are based on *F*, with *F* set to zero for negative *F*^2^. The threshold expression of *F*^2^ > σ(*F*^2^) is used only for calculating *R*-factors(gt) *etc*. and is not relevant to the choice of reflections for refinement. *R*-factors based on *F*^2^ are statistically about twice as large as those based on *F*, and *R*- factors based on ALL data will be even larger.

### Fractional atomic coordinates and isotropic or equivalent isotropic displacement parameters (Å^2^)

**Table:**

	*x*	*y*	*z*	*U*_iso_*/*U*_eq_	Occ. (<1)
O1	0.2493 (2)	0.69510 (15)	0.57618 (13)	0.0462 (4)	
N1	0.25327 (19)	0.49053 (16)	0.47345 (13)	0.0306 (3)	
C1	0.2670 (3)	0.4283 (2)	0.35441 (16)	0.0355 (4)	
H1A	0.3925	0.4604	0.3057	0.043*	
H1B	0.1605	0.4711	0.2977	0.043*	
C2	0.2505 (3)	0.2437 (2)	0.40071 (17)	0.0371 (4)	
H2A	0.1365	0.2019	0.3654	0.045*	
H2B	0.3686	0.1912	0.3739	0.045*	
C3	0.2275 (2)	0.21325 (19)	0.54586 (16)	0.0293 (4)	
C4	0.2056 (2)	0.0682 (2)	0.63687 (17)	0.0328 (4)	
H4	0.2030	−0.0309	0.6101	0.039*	
C5	0.1872 (2)	0.0670 (2)	0.76852 (17)	0.0331 (4)	
C6	0.1927 (2)	0.2141 (2)	0.80423 (16)	0.0340 (4)	
H6	0.1817	0.2138	0.8936	0.041*	
C7	0.2137 (2)	0.3621 (2)	0.71377 (16)	0.0320 (4)	
H7	0.2165	0.4614	0.7403	0.038*	
C8	0.2304 (2)	0.35962 (19)	0.58388 (15)	0.0278 (4)	
C9	0.2613 (2)	0.6503 (2)	0.47457 (18)	0.0337 (4)	
C10	0.2848 (3)	0.7693 (2)	0.34518 (19)	0.0404 (4)	
H10A	0.2923	0.8797	0.3582	0.061*	
H10B	0.1734	0.7598	0.2940	0.061*	
H10C	0.4042	0.7460	0.2992	0.061*	
C11	0.1537 (3)	−0.0898 (2)	0.86977 (18)	0.0422 (5)	
H11A	0.0257	−0.1352	0.8581	0.051*	
H11B	0.1485	−0.0653	0.9563	0.051*	
C12	0.3060 (3)	−0.2180 (2)	0.86693 (18)	0.0400 (4)	
H12A	0.3065	−0.2493	0.7859	0.048*	
Cl1	0.5409 (4)	−0.1512 (3)	0.8927 (2)	0.0525 (4)	0.50
C13	0.245 (2)	−0.3659 (13)	0.9768 (12)	0.100 (5)	0.50
H13A	0.2548	−0.3380	1.0605	0.150*	0.50
H13B	0.3311	−0.4566	0.9725	0.150*	0.50
H13C	0.1111	−0.3970	0.9672	0.150*	0.50
Cl1B	0.2300 (4)	−0.4048 (3)	0.97881 (18)	0.0532 (4)	0.50
C13B	0.4978 (16)	−0.1668 (14)	0.9079 (13)	0.102 (5)	0.50
H13D	0.5386	−0.0621	0.8516	0.152*	0.50
H13E	0.5958	−0.2483	0.9009	0.152*	0.50
H13F	0.4834	−0.1567	0.9977	0.152*	0.50

### Atomic displacement parameters (Å^2^)

**Table:**

	*U*^11^	*U*^22^	*U*^33^	*U*^12^	*U*^13^	*U*^23^
O1	0.0608 (9)	0.0299 (7)	0.0505 (8)	0.0026 (6)	−0.0098 (6)	−0.0135 (6)
N1	0.0303 (7)	0.0277 (7)	0.0333 (7)	0.0010 (5)	−0.0030 (6)	−0.0053 (6)
C1	0.0388 (9)	0.0357 (9)	0.0312 (9)	0.0025 (7)	−0.0018 (7)	−0.0063 (7)
C2	0.0447 (10)	0.0343 (9)	0.0334 (9)	−0.0024 (7)	0.0009 (7)	−0.0106 (7)
C3	0.0261 (8)	0.0299 (8)	0.0328 (9)	0.0017 (6)	−0.0018 (6)	−0.0092 (7)
C4	0.0309 (8)	0.0268 (8)	0.0409 (10)	0.0031 (6)	−0.0008 (7)	−0.0087 (7)
C5	0.0260 (8)	0.0341 (9)	0.0357 (9)	0.0046 (7)	−0.0005 (7)	−0.0016 (7)
C6	0.0304 (9)	0.0420 (10)	0.0293 (9)	0.0067 (7)	−0.0021 (7)	−0.0075 (7)
C7	0.0287 (8)	0.0330 (9)	0.0366 (9)	0.0045 (7)	−0.0044 (7)	−0.0122 (7)
C8	0.0218 (7)	0.0279 (8)	0.0335 (9)	0.0024 (6)	−0.0033 (6)	−0.0063 (6)
C9	0.0262 (8)	0.0279 (8)	0.0473 (10)	0.0011 (6)	−0.0070 (7)	−0.0081 (7)
C10	0.0332 (9)	0.0302 (9)	0.0534 (11)	0.0003 (7)	−0.0039 (8)	−0.0005 (8)
C11	0.0401 (10)	0.0401 (10)	0.0408 (10)	0.0038 (8)	0.0034 (8)	0.0006 (8)
C12	0.0495 (11)	0.0321 (9)	0.0361 (10)	0.0033 (8)	−0.0037 (8)	−0.0034 (7)
Cl1	0.0513 (8)	0.0619 (10)	0.0492 (7)	0.0144 (6)	−0.0190 (6)	−0.0190 (7)
C13	0.118 (9)	0.030 (6)	0.136 (8)	0.027 (5)	0.001 (5)	0.009 (4)
Cl1B	0.0777 (10)	0.0273 (10)	0.0482 (8)	0.0008 (7)	−0.0027 (6)	0.0036 (5)
C13B	0.081 (7)	0.049 (5)	0.161 (10)	0.028 (4)	−0.026 (6)	0.006 (5)

### Geometric parameters (Å, °)

**Table:**

O1—C9	1.227 (2)	C7—H7	0.9500
N1—C9	1.360 (2)	C9—C10	1.506 (3)
N1—C8	1.417 (2)	C10—H10A	0.9800
N1—C1	1.482 (2)	C10—H10B	0.9800
C1—C2	1.531 (2)	C10—H10C	0.9800
C1—H1A	0.9900	C11—C12	1.517 (3)
C1—H1B	0.9900	C11—H11A	0.9900
C2—C3	1.505 (2)	C11—H11B	0.9900
C2—H2A	0.9900	C12—C13B	1.511 (10)
C2—H2B	0.9900	C12—C13	1.543 (10)
C3—C4	1.379 (2)	C12—Cl1	1.774 (3)
C3—C8	1.395 (2)	C12—Cl1B	1.804 (3)
C4—C5	1.398 (2)	C12—H12A	0.9604
C4—H4	0.9500	C13—H13A	0.9800
C5—C6	1.389 (3)	C13—H13B	0.9800
C5—C11	1.513 (2)	C13—H13C	0.9800
C6—C7	1.395 (2)	C13B—H13D	0.9800
C6—H6	0.9500	C13B—H13E	0.9800
C7—C8	1.387 (2)	C13B—H13F	0.9800
			
C9—N1—C8	125.79 (15)	H10A—C10—H10B	109.5
C9—N1—C1	124.26 (14)	C9—C10—H10C	109.5
C8—N1—C1	109.95 (13)	H10A—C10—H10C	109.5
N1—C1—C2	105.54 (13)	H10B—C10—H10C	109.5
N1—C1—H1A	110.6	C5—C11—C12	114.95 (15)
C2—C1—H1A	110.6	C5—C11—H11A	108.5
N1—C1—H1B	110.6	C12—C11—H11A	108.5
C2—C1—H1B	110.6	C5—C11—H11B	108.5
H1A—C1—H1B	108.8	C12—C11—H11B	108.5
C3—C2—C1	104.39 (13)	H11A—C11—H11B	107.5
C3—C2—H2A	110.9	C13B—C12—C11	110.8 (5)
C1—C2—H2A	110.9	C13B—C12—C13	103.1 (7)
C3—C2—H2B	110.9	C11—C12—C13	106.5 (6)
C1—C2—H2B	110.9	C13B—C12—Cl1	7.7 (6)
H2A—C2—H2B	108.9	C11—C12—Cl1	112.22 (16)
C4—C3—C8	120.53 (15)	C13—C12—Cl1	109.3 (6)
C4—C3—C2	129.25 (15)	C13B—C12—Cl1B	107.8 (5)
C8—C3—C2	110.22 (14)	C11—C12—Cl1B	109.45 (16)
C3—C4—C5	120.04 (15)	C13—C12—Cl1B	7.6 (6)
C3—C4—H4	120.0	Cl1—C12—Cl1B	113.43 (15)
C5—C4—H4	120.0	C13B—C12—H12A	117.2
C6—C5—C4	118.45 (15)	C11—C12—H12A	109.8
C6—C5—C11	120.51 (16)	C13—C12—H12A	108.8
C4—C5—C11	120.99 (16)	Cl1—C12—H12A	110.2
C5—C6—C7	122.45 (16)	Cl1B—C12—H12A	101.2
C5—C6—H6	118.8	C12—C13—H13A	109.5
C7—C6—H6	118.8	C12—C13—H13B	109.5
C8—C7—C6	117.80 (15)	H13A—C13—H13B	109.5
C8—C7—H7	121.1	C12—C13—H13C	109.5
C6—C7—H7	121.1	H13A—C13—H13C	109.5
C7—C8—C3	120.73 (15)	H13B—C13—H13C	109.5
C7—C8—N1	129.36 (15)	C12—C13B—H13D	109.5
C3—C8—N1	109.90 (14)	C12—C13B—H13E	109.5
O1—C9—N1	121.52 (16)	H13D—C13B—H13E	109.5
O1—C9—C10	121.67 (16)	C12—C13B—H13F	109.5
N1—C9—C10	116.80 (16)	H13D—C13B—H13F	109.5
C9—C10—H10A	109.5	H13E—C13B—H13F	109.5
C9—C10—H10B	109.5		
			
C9—N1—C1—C2	−179.87 (15)	C4—C3—C8—N1	−179.69 (14)
C8—N1—C1—C2	−0.04 (18)	C2—C3—C8—N1	0.25 (18)
N1—C1—C2—C3	0.17 (17)	C9—N1—C8—C7	−0.8 (3)
C1—C2—C3—C4	179.67 (16)	C1—N1—C8—C7	179.34 (16)
C1—C2—C3—C8	−0.26 (18)	C9—N1—C8—C3	179.70 (14)
C8—C3—C4—C5	−0.4 (2)	C1—N1—C8—C3	−0.13 (18)
C2—C3—C4—C5	179.70 (16)	C8—N1—C9—O1	0.2 (3)
C3—C4—C5—C6	−0.4 (2)	C1—N1—C9—O1	−179.96 (16)
C3—C4—C5—C11	177.16 (15)	C8—N1—C9—C10	−179.55 (14)
C4—C5—C6—C7	0.7 (2)	C1—N1—C9—C10	0.3 (2)
C11—C5—C6—C7	−176.83 (15)	C6—C5—C11—C12	−125.81 (18)
C5—C6—C7—C8	−0.3 (2)	C4—C5—C11—C12	56.7 (2)
C6—C7—C8—C3	−0.4 (2)	C5—C11—C12—C13B	68.4 (6)
C6—C7—C8—N1	−179.86 (15)	C5—C11—C12—C13	179.9 (6)
C4—C3—C8—C7	0.8 (2)	C5—C11—C12—Cl1	60.3 (2)
C2—C3—C8—C7	−179.27 (14)	C5—C11—C12—Cl1B	−172.81 (15)

### Hydrogen-bond geometry (Å, °)

**Table:**

*D*—H···*A*	*D*—H	H···*A*	*D*···*A*	*D*—H···*A*
C4—H4···O1^i^	0.95	2.45	3.388 (2)	168.
C12—H12A···O1^i^	0.96	2.44	3.388 (2)	169.

Symmetry codes: (i) *x*, *y*−1, *z*.

## References

[bb1] Asselin, A. A., Humber, L. G., Crocilla, D., Oshiro, G., Wojdan, A., Grimes, D., Heaslip, R. J., Rimele, T. J. & Shaw, C. C. (2000). *J. Med. Chem.* **29**, 1009–1015. 10.1021/jm00156a019 3012084

[bb2] Bremner, J. B., Coban, B., Griffith, G., Groenewoud, K. M. & Yates, B. F. (2000). *Bioorg. Med. Chem.* **8**, 201–214. 10.1016/s0968-0896(99)00263-1 10968279

[bb3] Bruker (2001). *SMART* Bruker AXS Inc., Madison, Wisconsin, USA.

[bb4] Bruker (2003). *SAINT-Plus* Bruker AXS Inc., Madison, Wisconsin, USA.

[bb5] Burnett, M. N. & Johnson, C. K. (1996). *ORTEPIII*, Report ORNL-6895. Oak Ridge National Laboratory, Tennessee, USA.

[bb6] Elworthy, T. R., Ford, A. P., Bantle, G. W. & Morgans, D. J. (1997). *J. Med. Chem.* **40**, 2674–2687. 10.1021/jm970166j 9276013

[bb7] Farrugia, L. J. (1997). *J. Appl. Cryst.* **30**, 565.

[bb8] Moreno, M. M. T., Santos, R. H. A., Gambardella, M. T. P., Camargo, A. J., da Silva, A. B. F. & Trsic, M. (1998). *Struct. Chem.* **9**, 365–373.

[bb13] Sheldrick, G. M. (2008*a*). *SADABS.* University of Göttingen, Germany

[bb9] Sheldrick, G. M. (2008*b*). *Acta Cryst.* A**64**, 112–122. 10.1107/S0108767307043930 18156677

[bb10] Sorbera, L. A., Caster, J. & Silvestre, J. S. (2001). *Drugs Fut.* **26**, 553–555.

[bb11] Spek, A. L. (2009). *Acta Cryst.* D**65**, 148–155. 10.1107/S090744490804362X PMC2631630 19171970

[bb12] Wang, Z., Wan, W., Jiang, H. & Hao, J. (2007). *J. Org. Chem.* **72**, 9364–9367. 10.1021/jo701566v 17973530

